# Minimum surgical volume to ensure 5‐year survival probability for six cancer sites in Japan

**DOI:** 10.1002/cam4.4999

**Published:** 2022-07-07

**Authors:** Sumiyo Okawa, Takahiro Tabuchi, Toshitaka Morishima, Kayo Nakata, Shihoko Koyama, Satomi Odani, Isao Miyashiro

**Affiliations:** ^1^ Cancer Control Center Osaka International Cancer Institute Osaka Japan; ^2^ Institute for Global Health Policy Research Bureau of International Health Cooperation, National Center for Global Health and Medicine Tokyo Japan

**Keywords:** clinical guidelines, epidemiology, surgery, survival

## Abstract

**Background:**

In Japan, the government designates hospitals specialized in cancer care, requiring them to perform 400 surgeries annually without requiring surgical volume per cancer site. This study aimed to estimate the site‐specific minimum surgical volume per year based on its associations with 5‐year survival probability.

**Methods:**

The data of 64,402 patients who had undergone surgery for six types of cancers (including esophageal, stomach, colorectal, pancreatic, lung, and breast cancers) at designated cancer care hospitals in Osaka between 2007 and 2011 were analyzed. The hospitals were categorized by the average annual surgical volume per cancer type (e.g., 0–4, 5–9, 10–14…). We estimated the adjusted 5‐year survival probability per surgical volume category using multivariable Cox proportional hazard regression. Furthermore, we identified inflection points for the trend of adjusted survival probability per increase of five surgical volumes using the joinpoint regression model and considered them as the suggested minimum surgical volume.

**Results:**

The estimated minimum surgical volumes were 35–39, 20–25, 25–29, 10–14, 10–14, and 25–29 for esophageal, stomach, colorectal, pancreatic, lung, and breast cancers, respectively. The percentage change in the adjusted 5‐year survival probability per increase of five surgical volumes before and after the suggested surgical volume were +2.23 and +0.39 for the esophagus, +9.68 and +0.34 for the stomach, +8.11 and +0.05 for the colorectum, +3.82 and +0.87 for the pancreas, +9.46 and +0.23 for the lung, and +1.27 and +0.03 for the breast.

**Conclusions:**

The suggested surgical volume based on the association with survival probability varies with cancer sites, some of which are close to the existing surgical volume standards used in Japan. These evidence‐based minimum surgical volumes may help improve the quality of cancer surgeries.

## Graphical abstract

Designated cancer care hospitals are required to perform 400 surgeries annually in Japan, without requiring surgical volume per cancer site. This study estimated minimum surgical volume to be performed by designated cancer care hospitals based on the association between annual surgical volume and adjusted 5‐year survival probability for the following six cancer sites: 35‐39, 20‐25, 25‐29, 10‐14, 10‐14, and 25‐29 surgeries per year for esophageal, stomach, colorectal, pancreatic, lung, and breast cancers, respectively. These evidence‐based minimum surgical volumes may help improve the quality of cancer surgeries in designated cancer care hospitals in Japan.

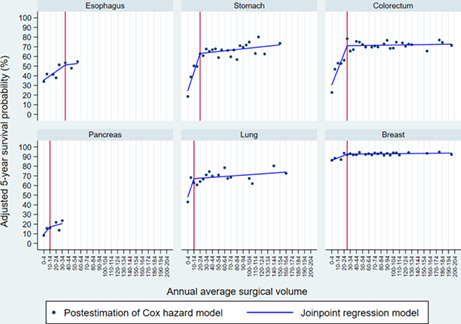

## 1 INTRODUCTION

Cancer is associated with the growing burden of disease in public health. Globally, 18.1 million people had cancer, and 9.6 million died from the disease in 2018.^1^ Cancer control to reduce the burden of cancer mainly comprises primary prevention, screening, early diagnosis, treatment, survivorship, and palliative care.^1^ Of these interventions, treatment is largely responsible for patient outcomes. The centralization of patients at medical facilities equipped with better infrastructure, resources, knowledge, and experience is a common strategy to improve the quality of care, patient outcomes, and financial efficiency.^2^, ^3^ The concept of centralization stems from the evidence that higher‐volume hospitals are associated with better patient outcomes.^4^ For example, in cancer treatment, patients treated in high‐volume hospitals showed higher 5‐year survival rates^5^, ^6^ and lower mortality rates^7^ than those treated in low‐volume hospitals. Although the underlying mechanism of this association has not been established,^8^ high‐volume hospitals may be more capable of practicing specific surgical procedures and intensive care and managing postoperative complications than low‐volume hospitals.^9^, ^10^

Several countries define the minimum surgical volume standards as a part of the centralization strategy.^2^, ^11^ For example, of 22 European and North American countries, more than half have surgical volume standards for esophageal, pancreatic, liver, or rectal cancers, but the cutoffs of volume standards vary with cancer site and country.^2^ Some studies have reported that hospitals that meet surgical volume standards report lower mortality than those that do not meet the standards.^12^, ^13^ In contrast, some studies found no association, calling for the need to identify the appropriate requirements.^14^, ^15^ This implies that the surgical volume standard should be defined according to the health system, regulation, cancer burden,^11^ or training system of the surgical experts in local settings, and some studies have investigated the minimum surgical volumes for specific cancer sites based on the associations with patient outcomes.^16^, ^17^, ^18^

In Japan, cancer has been the leading cause of death since 1981, accounting for 27% of all‐cause deaths in 2018.^19^ The national government addresses nationwide cancer control, namely the Basic Plan to Promote Cancer Control programs,^20^ and developed cancer care system which placed Designated Cancer Care Hospitals (DCCHs) at the center of cancer treatment and cares to ensure patients with cancer access high‐quality cancer care regardless of where they live.^21^ As of 2021, 405 DCCHs were designated by the national government (i.e., the national DCCHs) across the country.^22^ In addition, prefectural governments designate prefectural DCCHs to strengthen the local cancer care system.^23^ Both national and prefectural DCCHs need to meet various standards for structure, resource, and clinical practice, including annual surgical volume for all cancers.^24^, ^25^ However, surgical volume standards per cancer site are not defined for DCCHs. Meanwhile, medical societies or associations define surgical volume standards to certify hospitals as high‐standard medical or training facilities. For example, the required surgical volumes are ≥50 esophageal surgeries within 5 years,^26^ ≥60 surgical gastrectomies for gastric cancer within the last 3 years,^27^ ≥100 colorectal and anal surgeries per year,^28^ average ≥ 20 pancreatic cancer cases per year registered in the national clinical database within the last 5 years,^29^ average ≥ 150 thoracic surgeries per year in the last 3 years to certify flagship facilities for specialized training, an average of ≥25 thoracic surgeries per year in the last 3 year to accredit collaboration facility for specialized training,^30^ and ≥30 surgeries for breast cancers annually.^31^

The association between high surgical volume and better patient outcomes is known in Japan,^32^, ^33^, ^34^, ^35^ and minimum surgical volume thresholds for solid cancers based on the association with patient survival probability have been previously reported.^36^ However, the minimum surgical volume for specific cancer sites has not been examined based on the volume–outcome relationship. Therefore, this study aimed to identify the minimum surgical volume per cancer site based on the association between surgical volume and adjusted 5‐year survival probability.

## 2 MATERIALS AND METHODS

### 2.1 Study design, setting, and data source

This retrospective cohort study used a population‐based cancer registry in Osaka, Japan, named Osaka Cancer Registry.^37^ The registry has been operated since 1962, and data on newly diagnosed patients with cancer across the Osaka prefecture were collected. Furthermore, the registry routinely follows up on the vital status of patients at 3, 5, and 10 years after the diagnosis of cancer by reviewing death certificates and official resident registries.^37^ The data quality of the registry is considered high as the data have been used by the International Agency for Research on Cancer Incidence in Five Continents, volumes III to XI.^38^ The registry database comprises patient information on sex, age at diagnosis, date of diagnosis and death, cancer site, cancer stage, and treatment that a patient received for cancer (i.e., open surgery, endoscopic surgery, endoscopic resection, chemotherapy, radiation therapy), the existence of residual tumors after surgery, and medical facility code where a patient received the first diagnosis of cancer and underwent primary treatment, respectively. The registry database does not have information on socioeconomic characteristics, comorbidity status, performance status, details of treatment for cancer and other diseases, and causes of death.

### 2.2 Study sample

The inclusion criteria were as follows: diagnosis of cancers of the esophagus (C15 in the International Classification of Diseases 10th edition), stomach (C16), colorectum (C18, C19, and C20), pancreas (C25), lung (C33 and C34), or breast (C50) between 2007 and 2011 for patients living in Osaka at the time of diagnosis and undergoing open or endoscopic surgery in the 66 hospitals that were the DCCHs for adult cancers as of 2020.^39^ We targeted six cancer sites because stomach, colorectal, breast, and lung cancers are the most prevalent cancers in Osaka, accounting for 63% of all cancers.^40^ DCCHs were responsible for offering high‐quality cancer treatment and care to patients with these common cancers within their residential areas.^21^ Furthermore, surgeries for esophageal, pancreatic, and lung cancers are considered high‐risk procedures, and the surgical volume standard for these cancers was defined by the US’ patient safety organization “Leapfrog.”^41^ DCCHs covered approximately 86% of surgeries for solid cancers in Osaka between 2010 and 2012.^40^ Of these, three hospitals were DCCHs for only lung cancer, which were excluded from the analyses for cancers other than lung cancer. Patients aged ≥15 years were included in the calculation of the annual surgical volume per DCCH. Survival analysis was conducted for patients aged 15–84 years because old age is a strong determinant for the choice of hospital^42^ and treatment outcomes. Patients with unknown survival status at 5 years from diagnosis, those with a survival duration of zero days, or those with a lack of information on sex or cancer stage were excluded from the survival analysis.

### 2.3 Outcome variable: Survival within 5 years from the diagnosis of cancer

The study outcome was defined as survival within 5 years from the cancer diagnosis. The monitoring of a patient was terminated on the date of death or censored at 5 years after the diagnosis.

### 2.4 Explanatory variable: Annual average surgical volume between 2007 and 2011

Surgical volume was defined as the annual average number of open or endoscopic surgeries in a DCCH involving patients aged ≥15 years between 2007 and 2011. The surgical volumes were calculated for esophageal, stomach, colorectal, pancreatic, lung, and breast cancers and categorized by an increase of five annual surgical volumes (e.g., 0–4, 5–9, 10–14…). The patients were sorted according to the surgical volume category of the DCCHs.

### 2.5 Statistical analysis

First, the frequencies and percentages of the basic characteristics of patients and the distributions of DCCHs and patients per surgical volume category (i.e., the number of patients, patient mean age, and percentage of patients with localized cancer stage) were calculated for each cancer site. Subsequently, the 5‐year mortality hazard ratios (HRs) and 95% confidence intervals (CIs) of the surgical volume categories were estimated for each cancer site using the multivariable Cox proportional hazard regression model. The proportional hazards assumption was visually evaluated using a log–log survival plot. The adjustment variables in the model included sex (men, women), age category (15–39, 40–49, 50–59, 60–69, 70–79, 80–84 years), cancer stage (localized, regional, distant, unknown), surgical procedure types (open surgery, endoscopic surgery), existence of residual tumors after surgery (no residual tumor, residual tumor, unknown), reception of chemotherapy or hormone therapy (received, not received, unknown), reception of radiation therapy (received, not received, unknown), residential area corresponding to the secondary medical administration zone (areas A, B, C, D, E, F, G, H), and year of diagnosis (2007, 2008, 2009, 2010, 2011). These variables were selected based on previous studies.^16^, ^18^, ^36^ Further, the 95% CIs of the HRs were adjusted using robust estimators of variance to control for cluster correlations of patients nested in the same treatment hospital. Multicollinearity was assessed based on the variance inflation factor.

The minimum surgical volume was estimated using the following procedures. First, we estimated the adjusted 5‐year survival probability per surgical volume category using the postestimation function of the multivariable Cox proportional hazard model, for which the adjusted survival probability was calculated based on the mean value of each explanatory variable for the overall estimated sample.^43^ We used the joinpoint regression model to identify the suggested minimum surgical volume.^44^ The details of the model have been described elsewhere.^45^ In brief, the joinpoint regression model analyzes the change in trend over consecutive segments of the explanatory variable. The model identifies inflection points (i.e., joinpoints) at which a significant increase or decrease in the trend exists. The model also calculates the slope of the trend below and above the joinpoint based on the best‐fitting model. We applied this model to identify joinpoints at which the trend of adjusted 5‐year survival probability per increase of five surgery changes based on the assumption that the surgical volume is positively associated with patient outcomes until it reaches a certain volume and the association plateaus.^2^ We considered joinpoints as the suggested minimum surgical volume. Furthermore, we calculated the slopes of associations between surgical volume and adjusted 5‐year survival probability below and above the suggested surgical volume, respectively. The value of the slope refers to the percentage change in the adjusted 5‐year survival probability per an increase of five surgeries per year.

Besides the aforementioned main analyses, we performed sensitivity analyses in which the definition of annual surgical volume was the same as the main analysis. However, patients with metastatic or unknown cancer stages were excluded from the survival analysis.

Statistical significance was considered at *p* < 0.05. The Stata 15.1 statistical software package (Stata Corp, College Station, Texas, USA) was used for descriptive analysis and the Cox proportional hazard model. The Joinpoint Regression Program, version 4.8.0.1 (Statistical Research and Applications Branch, National Cancer Institute, Calverton, USA), was used for the joinpoint regression model.

## 3 RESULTS

### 3.1 Basic characteristics of the patient included in the analysis

Table 1 shows the distributions of the basic characteristics of patients per cancer site. The number of patients included in the analysis was 2211, 15,040, 21,545, 2035, 8872, and 14,699 for esophageal, stomach, colorectal, pancreatic, lung, and breast cancers, respectively. Regarding the cancer stage, the localized stage accounted for the highest proportions of stomach, colorectal, lung, and breast cancers, whereas the regional stage accounted for the highest proportions of esophageal and pancreatic cancers. More than 76% of patients with esophageal, stomach, colorectal, lung, and breast cancers had no residual tumors after surgery; however, 33.5% of patients with pancreatic cancers had residual tumors.

**TABLE 1:** Basic characteristics of patients

	Esophagus		Stomach		Colorectum		Pancreas		Lung		Breast	
	*n*	(%)	*n*	(%)	*n*	(%)	*N*	(%)	*n*	(%)	*n*	(%)
Total	2211		15,040		21,545		2035		8872		14,699	
Sex												
Men	1820	(82.3)	10,496	(69.8)	12,655	(58.7)	1144	(56.2)	5659	(63.8)	92	(0.6)
Women	391	(17.7)	4544	(30.2)	8890	(41.3)	891	(43.8)	3213	(36.2)	14,607	(99.4)
Age												
15–39	6	(0.3)	212	(1.4)	269	(1.2)	19	(0.9)	75	(0.8)	958	(6.5)
40–49	70	(3.2)	630	(4.2)	822	(3.8)	67	(3.3)	270	(3.0)	3059	(20.8)
50–59	439	(19.9)	2242	(14.9)	3055	(14.2)	253	(12.4)	1089	(12.3)	3339	(22.7)
60–69	1006	(45.5)	5111	(34.0)	7286	(33.8)	747	(36.7)	3285	(37.0)	4125	(28.1)
70–79	618	(28.0)	5393	(35.9)	7945	(36.9)	800	(39.3)	3510	(39.6)	2561	(17.4)
80–84	72	(3.3)	1452	(9.7)	2168	(10.1)	149	(7.3)	643	(7.2)	657	(4.5)
Stage												
Localized	587	(26.5)	7423	(49.4)	9482	(44.0)	282	(13.9)	5633	(63.5)	9693	(65.9)
Regional	1320	(59.7)	5395	(35.9)	7986	(37.1)	1275	(62.7)	2605	(29.4)	4488	(30.5)
Distant	205	(9.3)	2008	(13.4)	3741	(17.4)	425	(20.9)	478	(5.4)	250	(1.7)
Unknown	99	(4.5)	214	(1.4)	336	(1.6)	53	(2.6)	156	(1.8)	268	(1.8)
Surgical procedure type												
Open surgery	1794	(81.1)	11,473	(76.3)	14,256	(66.2)	2000	(98.3)	4260	(48.0)	14,690	(99.9)
Endoscopic surgery	417	(18.9)	3567	(23.7)	7289	(33.8)	35	(1.7)	4612	(52.0)	9	(0.1)
Existence of residual tumors												
No residual tumor	1711	(77.4)	11,623	(77.3)	16,905	(78.5)	1224	(60.1)	6819	(76.9)	12,609	(85.8)
Residual tumor	374	(16.9)	2400	(16.0)	3106	(14.4)	681	(33.5)	1368	(15.4)	1230	(8.4)
Unknown	126	(5.7)	1017	(6.8)	1534	(7.1)	130	(6.4)	685	(7.7)	860	(5.9)
Chemo/Hormone therapy												
Received	1240	(56.1)	4772	(31.7)	7887	(36.6)	1149	(56.5)	2505	(28.2)	10,408	(70.8)
Not received	960	(43.4)	9998	(66.5)	13,162	(61.1)	863	(42.4)	6179	(69.6)	3950	(26.9)
Unknown	11	(0.5)	270	(1.8)	496	(2.3)	23	(1.1)	188	(2.1)	341	(2.3)
Radiation therapy												
Received	381	(17.2)	52	(0.3)	453	(2.1)	203	(10.0)	465	(5.2)	5383	(36.6)
Not received	1822	(82.4)	14,766	(98.2)	20,698	(96.1)	1797	(88.3)	8234	(92.8)	9153	(62.3)
Unknown	8	(0.4)	222	(1.5)	394	(1.8)	35	(1.7)	173	(1.9)	163	(1.1)
Residential area												
Area A	653	(29.5)	4431	(29.5)	6959	(32.3)	577	(28.4)	2659	(30.0)	4315	(29.4)
Area B	265	(12.0)	1782	(11.8)	2830	(13.1)	242	(11.9)	857	(9.7)	1846	(12.6)
Area C	184	(8.3)	1266	(8.4)	1713	(8.0)	134	(6.6)	924	(10.4)	1204	(8.2)
Area D	275	(12.4)	1707	(11.3)	2014	(9.3)	276	(13.6)	940	(10.6)	1540	(10.5)
Area E	208	(9.4)	1598	(10.6)	2357	(10.9)	192	(9.4)	866	(9.8)	1460	(9.9)
Area F	166	(7.5)	1058	(7.0)	1267	(5.9)	154	(7.6)	759	(8.6)	984	(6.7)
Area G	249	(11.3)	1476	(9.8)	2057	(9.5)	217	(10.7)	1006	(11.3)	1624	(11.0)
Area H	211	(9.5)	1722	(11.4)	2348	(10.9)	243	(11.9)	861	(9.7)	1726	(11.7)
Years of diagnosis												
2007	442	(20.0)	3065	(20.4)	4101	(19.0)	364	(17.9)	1631	(18.4)	2837	(19.3)
2008	418	(18.9)	2911	(19.4)	3901	(18.1)	381	(18.7)	1553	(17.5)	2745	(18.7)
2009	440	(19.9)	2944	(19.6)	4224	(19.6)	382	(18.8)	1676	(18.9)	2737	(18.6)
2010	458	(20.7)	2969	(19.7)	4515	(21.0)	455	(22.4)	1894	(21.3)	3164	(21.5)
2011	453	(20.5)	3151	(21.0)	4804	(22.3)	453	(22.3)	2118	(23.9)	3216	(21.9)

### 3.2 Distributions of DCCHs and patients per surgical volume category

Tables S1 and S2 show the distribution of DCCHs and patients per surgical volume category (supporting information). The number of DCCH where patients with esophageal, stomach, colorectal, pancreatic, lung, and breast cancers underwent surgery was 57, 63, 63, 62, 64, and 62, respectively. The DCCHs were classified eight surgical volume categories (range: 0–4 to 55–59) for esophageal cancer, 24 (range: 0–4 to 150–154) for stomach cancer, 28 (range: 0–4 to 195–199) for colorectal cancer, 6 (range: 0–4 to 30–34) for pancreatic cancer, 17 (range: 0–4 to 160–164) for lung cancer, and 25 (range: 0–4 to 195–199) for breast cancer. Tables S3 and S4 shows patient mean age and percentage of patients with localized cancer stage per surgical category (supporting information).

### 3.3 Adjusted 5‐year mortality hazards by surgical volume

Table S5 shows the adjusted 5‐year mortality hazards of the surgical volume category and the adjusted 5‐year survival probability (supporting information). In the model, the category with the highest surgical volume was used as the reference group. The model appeared to meet the proportional hazard assumption, according to log–log survival plot testing. The adjustment variables had no multicollinearity according to the variance inflation factor. For esophageal cancers, the HRs were significantly higher for the surgical volume categories between 0–4 and 20–24. For stomach cancers, the HRs were significantly higher for the surgical volume categories between 0–4 and 40–44, 50–54 and 80–84, 90–94, 110–114, and 125–129 and significantly lower for 115–199. For colorectal cancers, the HRs were significantly higher for the surgical volume categories of 0–4, 5–9, 10–14, 20–24, 30–34, 95–99, and 155–159 and significantly lower for the categories of 25–29, 40–44, 90–94, and 175–179. For pancreatic cancers, the HR was significantly higher for the surgical volume category of 0–4 only. For lung cancers, the HRs were significantly higher for the surgical volume categories of 0–4, between 10–14 and 25–29, 100–104, and 105–109 and significantly lower for the categories of 60–64 and 140–144. For breast cancer, the HRs were significantly higher for the surgical volume category of 5–9 and significantly lower for the 45–49, 80–84, 125–129, and 175–179 categories.

### 3.4 Minimum surgical volume based on the associations with the adjusted 5‐year survival probability

**FIGURE 1:**
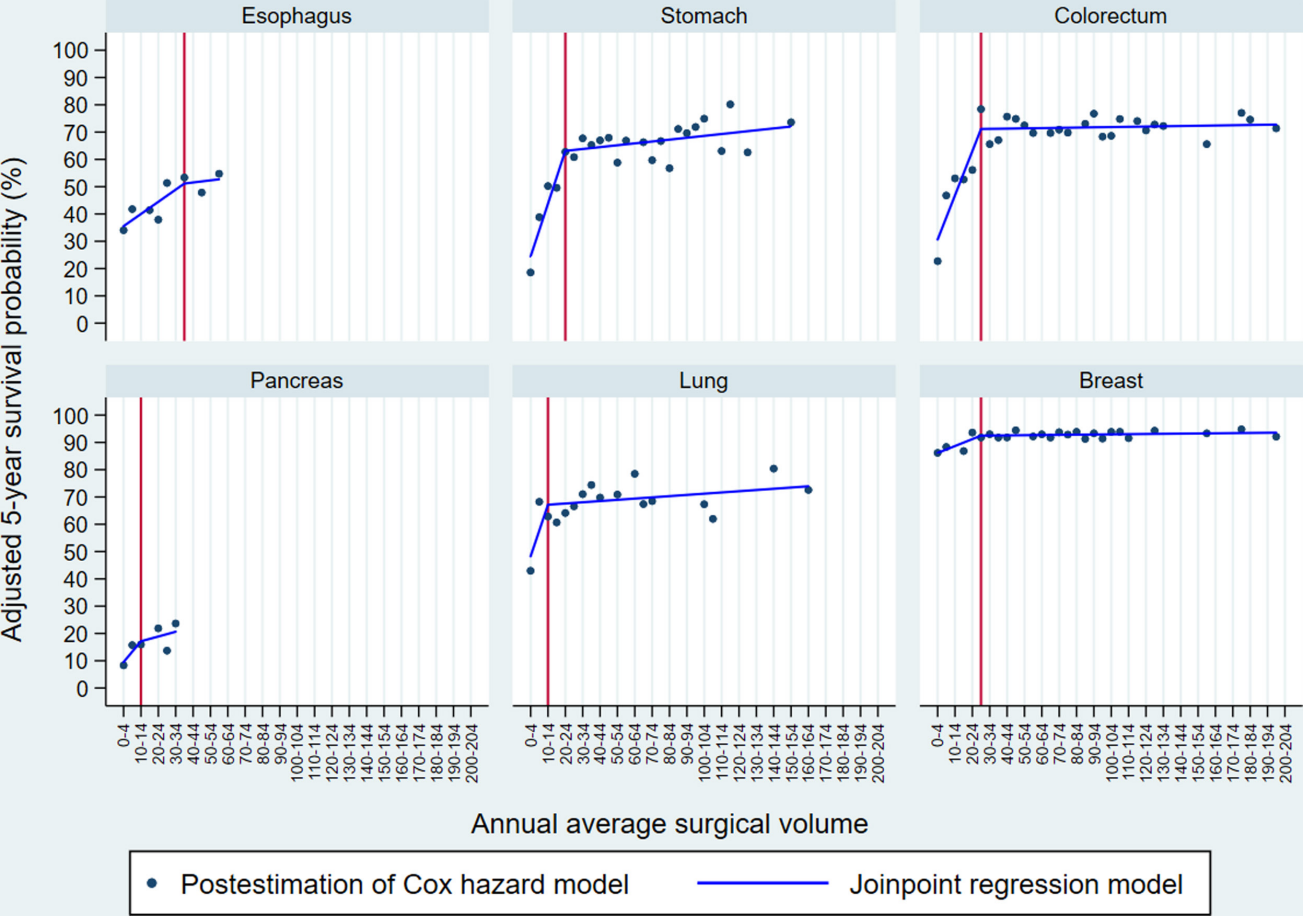
Trends of adjusted 5‐year survival probability per surgical volume category and the suggested minimum surgical volume. Note: We described the adjusted 5‐year survival probability per annual surgical volume category estimated by a multivariable Cox proportional hazard model. Based on the association between surgical volume category and adjusted 5‐year survival probability, we described the linear relationship and identified the minimum surgical volume using the joinpoint regression model. Red vertical lines indicate the suggested minimum surgical volume.

Figure 1 shows the trends of adjusted 5‐year survival probability per increase of five surgical volumes and their joinpoints, which we considered the suggested minimum surgical volume. The suggested surgical volumes were identified as 35–39, 20–24, 25–29, 10–14, 10–14, and 25–29 for esophageal, stomach, colorectal, pancreatic, lung, and breast cancers, respectively. The slope below and above the suggested surgical volume (by percent points), which indicates the extent of change in the adjusted 5‐year survival probability per increase of five surgeries, was +2.23 and + 0.39 for the esophagus, +9.68 and + 0.34 for the stomach, +8.11 and + 0.05 for the colorectum, +3.82 and + 0.87 for the pancreas, +9.46 and + 0.23 for the lung, and + 1.27 and + 0.03 for the breast.

Figure S1 shows the trends of adjusted 5‐year survival probability per increase of five surgical volumes and their joinpoints which excluded patients with metastatic or unknown cancer stages (supporting information). The suggested surgical volumes were identified as 35–39, 20–24, 25–29, 10–14, 30–34, and 20–24 for esophageal, stomach, colorectal, pancreatic, lung, and breast cancers, respectively.

## 4 DISCUSSION

This study examined the association between surgical volume and adjusted 5‐year survival probability for six selected cancers and estimated the suggested minimum surgical volume based on the association with survival probability among DCCHs in Osaka. Although DCCHs are specialized in cancer treatment and care, DCCHs with higher surgical volume appeared to show a higher adjusted 5‐year survival probability than those with lower volume, as several studies have reported the volume‐outcome relationship. To the best of our knowledge, this is one of the few studies^16^, ^17^, ^18^ that have identified the suggested minimum surgical volume per cancer site. The surgical volumes of the DCCHs showed a wide range per cancer site, with maximum surgical volumes of 55–59, 150–154, 195–199, 30–34, 160–164, and 195–199 for esophageal, stomach, colorectal, pancreatic, lung, and breast cancers, respectively. Within these ranges, the suggested surgical volumes were identified at 35–39, 20–24, 25–29, 10–14, 10–14, and 25–29 surgeries per year for esophageal, stomach, colorectal, pancreatic, lung, and breast cancers, respectively, after adjustment for patient characteristics. Upward trends in the adjusted 5‐year survival probability per increase in surgical volume were observed until the suggested surgical volume thresholds across the six cancer sites. After that, the trends nearly plateaued, as similar trends were observed in previous studies.^46^, ^47^ However, the slopes for the 5‐year survival probability per increase in surgical volume varied with cancer site. Further, there could be no specific minimum surgical volume identified if the slopes before and after the joinpoint were flat or constant (i.e., liner trend). Thus, various aspects should be considered for the application of the minimum surgical volume in policy and clinical settings, such as the slopes before and after the suggested minimum surgical volume, hazard ratios, and opinions of surgical specialists.

Of the six selected cancers assessed, the stomach, colorectal, lung, and breast cancers were the major cancers in Osaka. Our results showed that the slopes of the associations between surgical volume and the adjusted 5‐year survival probability for stomach, colorectal, and lung cancers showed steep trends until the suggested surgical volume, which flattened subsequently. This indicates that patients treated at lower‐volume DCCHs show lower survival probability, which suggests that monitoring and strengthening low‐volume DCCH function will be important to improve patient outcomes. This is because the DCCH system was developed to offer high‐quality cancer medicine to patients with these common cancers within their residential areas.^21^ To improve the performance of low‐volume DCCHs, availability of physician specialty training and intensive care units, availability of certain clinical services, staff‐patient ratio, and adherence to evidence‐based care should be considered, as these are prioritized in high‐volume hospitals.^8^ Breast cancers showed a minimum disparity in the adjusted 5‐year survival probability by surgical volume, and the slopes before and after the suggested surgical volume threshold were not steep, probably because the survival probability of breast cancer is higher than that of other cancers. Moreover, the effect of surgery on the survival probability of breast cancers may be relatively smaller than that of other cancers because chemotherapy and radiation therapy also play critical roles in treating breast cancers.

More than 90% and 80% of DCCHs that performed surgeries for esophageal and pancreatic cancers, respectively, reported less than the suggested surgical volume; 54% and 46% of patients with esophageal and pancreatic cancers were surgically treated at DCCHs with volumes below the suggested surgical volume, although the estimated joinpoint regression was less likely to be robust than that for other cancer sites because of the smaller sample, which requires careful interpretation. Despite this, our result shows that patients with these cancers are scattered across low‐volume DCCHs, although the number of patients was lower than that for other cancers. It is likely that many patients with esophageal and pancreatic cancers may first access a hospital near their places of residence, which may not necessarily be a DCCH with sufficient surgical volume. However, these hospitals may not necessarily have sufficient experience with esophageal and pancreatic surgeries. Furthermore, most patients were diagnosed with regional‐ or distant‐staged cancers, which were associated with poor prognoses. However, physicians or patients may not choose referrals to higher‐volume DCCHs. Similarly, in the United States and Germany, a high proportion of patients treated in hospitals that do not meet surgical volume standards have been reported.^12^, ^14^, ^48^ These findings suggest that centralizing patients with esophageal and pancreatic cancers at higher‐volume DCCHs and developing better surgical or non‐surgical treatment options will improve their survival probability, as the centralization policy has successfully improved patient outcomes with pancreatic cancer in the Netherlands^49^ and those with esophagogastric cancer in England.^50^ Meanwhile, centralization may increase travel distance to hospitals^51^ and create barriers for vulnerable patients to receive care in high‐volume hospitals.^52^ In Osaka, these would be less critical concerns because it has a well‐developed transportation system and 66 DCCHs in operation despite the small‐sized prefecture, and nearly, the entire population in the country is insured.^53^

We compared our suggested surgical volume with existing surgical volume standards by the Japanese medical societies or associations (Table 2). Regarding Japanese medical societies and associations, surgical volume standards for stomach, pancreatic, and breast cancer surgeries are used for credentialing hospitals, but those specific for esophageal, colorectal, and lung cancers are not available. The suggested minimum surgical volume of this study and the existing surgical volume standards per year were as follows: 20–24 versus 20 for stomach cancer,^27^ 10–14 versus 20 for pancreatic cancer,^29^ and 25–29 versus 30 for breast cancer,^31^ respectively. This demonstrates that the existing surgical volume standards are almost equal to or slightly higher than the suggested surgical volume.

**TABLE 2:** Minimum surgical volume suggested by this study and existing minimum volume standards per year

	This study	Japanese medical societies or associations	The US′s Leapfrog	German Cancer Society
Esophagus	35–39	10^a^	20^b^	–
Stomach	20–24	20^c^	–	–
Colorectum	25–29	100^d^	–	50^e^
Pancreas	10–14	20^f^	20^b^	12^g^
Lung	10–14	150, 25^h^	40^b^	75^i^
Breast	25–29	30^j^	–	–

^a^ ≥50 cases of esophageal surgery in 5 years (not specific to surgery for esophageal cancers).^26^

^b^ Leapfrog′s minimum surgical volume standard (per year).^41^

^c^ ≥60 cases of surgical gastrectomy for gastric cancers in the latest 3 years.^27^

^d^ ≥100 cases of colorectal and anal surgeries per year (not specific to surgery for colorectal cancers).^28^

^e^ ≥30 operative primary cases of colon cancers, ≥20 operative primary cases of rectal cancers per year.^54^

^f^ Average ≥20 cases of pancreatic cancer per year registered in the national clinical database for the last 5 years.^29^

^g^ ≥12 surgical primary cases of pancreatic cancers per year.^55^

^h^ Average ≥150 cases of thoracic surgery per year in the last 3 years for flagship facility for specialized training, average ≥25 cases of thoracic surgery per year in the last 3 years for collaboration facility for specialized training (not specific for lung cancers).^30^

^i^ ≥75 cases of lung cancer resections per year.^56^

^j^ ≥30 cases of surgery for breast cancers per year.^31^

The suggested minimum surgical volume was not necessarily consistent with the surgical volume standards in other settings. To investigate this, we compared our suggested surgical volume with the surgical volume standards used in the US and Germany (Table 2). Our suggested surgical volume and the standards of the US Leapfrog organization were as follows: 35–39 versus 20 for esophagus cancer,^41^ 10–14 versus 20 for pancreatic cancer,^41^ and 10–14 versus 40 for lung cancer.^41^ We also compared our suggested surgical volume with the surgical volume standards for the certified cancer center program of the German Cancer Society; our suggested surgical volume and the standards were as follows: 25–29 versus 50 for colorectal cancer (e.g., 30 colon and 20 rectal cancer surgeries),^54^ 10–14 versus 12 for pancreatic cancer,^55^ and 10–14 versus 75 for lung cancers.^56^ These comparisons showed that the extent of the similarity between our suggested surgical volume and the existing standards in the US and Germany varies by cancer site. These differences are not surprising because they imply that estimates of the minimum surgical volume are affected by incidence, prognosis, and other contextual factors in local settings; thus, a single surgical volume threshold is not necessarily suitable for all contexts.

This study had several limitations. First, the hazard ratios and survival probabilities may be biased because potential confounders that were unavailable in our cancer registry were not adjusted. They are socioeconomic characteristics, comorbidity, performance status, time from diagnosis to surgery, and any disease and treatment that a patient had after surgery for cancer. Other hospital characteristics (e.g., surgeon volume, human and material resources, or infrastructure) were not adjusted. Second, the cancer‐specific mortality rate could not be estimated because of the absence of information on the cause of death in the registry. Finally, the generalizability of the study findings is limited because the burden of each cancer and existing regulations and health systems may vary in other parts of the country and the world. However, Osaka prefecture has a high‐quality population‐based cancer registry and a well‐functioned designated cancer care hospital system. Thus, our study findings may reflect the situation of urban prefectures with well‐functioning designated cancer care hospital system. Further, future research that estimates minimum surgical volume using nationwide data will be worthwhile when the data become officially available.

In conclusion, this study identified and suggested the minimum surgical volume of hospitals designated for cancer care. The identified surgical volume varied with the cancer site. Although a surgical volume standard for all cancers has been applied to assess the performance of DCCHs, cancer site‐specific surgical volume standards may have clinical implications for improving the quality of surgery and reducing preventable complications and deaths. Further centralization of patients with esophageal and pancreatic cancers at high‐volume DCCHs should be encouraged. In addition, strengthening the capability of low‐volume DCCHs to treat common cancers is also important to ensure that all patients receive high‐quality treatment within their residential area and, consequently, improve their survival.

## AUTHOR CONTRIBUTIONS

SO and IM conceived and designed the study. SO conducted the statistical analysis. SO, TT, and MI interpreted the data. SO drafted the manuscript. All authors contributed to the critical revision of the manuscript and approved the final version of the manuscript. IM supervised the study.

## CONFLICT OF INTEREST

Dr. Isao Miyashiro is a member of the Cancer Registry Data Use Review Committee, which evaluates the confidentiality of study subjects. His work as a committee member does not affect any part of this study.

## ETHICS STATEMENT

Ethical approval was obtained from the Institutional Review Board of the Osaka International Cancer Institute (approval number: 19143, 19144) before conducting the study. We obtained the dataset with no personally identifiable information from the Osaka Cancer Registry and independently processed it according to the Act on the Promotion of Cancer Registries. Informed consent was not required for the patients.

## Supporting information


**Appendix S1** Supporting Information

## Data Availability

Data that support the findings of this study is available from Osaka Prefecture on reasonable request. (https://oici.jp/ocr/data/datause_guide.html)
