# Supplementary material for: Minimum surgical volume to ensure 5‐year survival probability for six cancer sites in Japan
Source: Cancer Med. 2022 Jul 7;12(2):1293–304. doi: 10.1002/cam4.4999 (PMC9883575; doi:10.1002/cam4.4999)
Supplement: Supplementary file 1 — Appendix S1 Supporting Information [file CAM4-12-1293-s001.docx]

**Table S1. Distribution of designated cancer care hospitals according to the surgical volume category**

| Surgical volume | Esophagus | |  | Stomach | |  | Colorectum | |  | Pancreas | |  | Lung | |  | Breast | |
| --- | --- | --- | --- | --- | --- | --- | --- | --- | --- | --- | --- | --- | --- | --- | --- | --- | --- |
|  | n | (%) |  | n | (%) |  | n | (%) |  | n | (%) |  | n | (%) |  | n | (%) |
| 0-4 | 39 | (68.4) |  | 1 | (1.6) |  | 1 | (1.6) |  | 34 | (54.8) |  | 21 | (32.8) |  | 9 | (14.5) |
| 5-9 | 9 | (15.8) |  | 4 | (6.4) |  | 3 | (4.8) |  | 16 | (25.8) |  | 3 | (4.7) |  | 7 | (11.3) |
| 10-14 |  |  |  | 2 | (3.2) |  | 2 | (3.2) |  | 7 | (11.3) |  | 4 | (6.3) |  |  |  |
| 15-19 | 2 | (3.5) |  | 6 | (9.5) |  | 2 | (3.2) |  |  |  |  | 5 | (7.8) |  | 2 | (3.2) |
| 20-24 | 2 | (3.5) |  | 4 | (6.4) |  | 1 | (1.6) |  | 2 | (3.2) |  | 8 | (12.5) |  | 3 | (4.8) |
| 25-29 | 1 | (1.8) |  | 5 | (7.9) |  | 1 | (1.6) |  | 1 | (1.6) |  | 3 | (4.7) |  | 3 | (4.8) |
| 30-34 |  |  |  | 3 | (4.8) |  | 4 | (6.4) |  | 2 | (3.2) |  | 4 | (6.3) |  | 8 | (12.9) |
| 35-39 | 1 | (1.8) |  | 4 | (6.4) |  | 3 | (4.8) |  |  |  |  | 1 | (1.6) |  | 2 | (3.2) |
| 40-44 |  |  |  | 6 | (9.5) |  | 2 | (3.2) |  |  |  |  | 2 | (3.1) |  | 3 | (4.8) |
| 45-49 | 1 | (1.8) |  | 3 | (4.8) |  | 2 | (3.2) |  |  |  |  |  |  |  | 1 | (1.6) |
| 50-54 |  |  |  | 3 | (4.8) |  | 3 | (4.8) |  |  |  |  | 2 | (3.1) |  |  |  |
| 55-59 | 2 | (3.5) |  | 3 | (4.8) |  | 4 | (6.4) |  |  |  |  |  |  |  | 2 | (3.2) |
| 60-64 |  |  |  |  |  |  |  |  |  |  |  |  | 1 | (1.6) |  | 2 | (3.2) |
| 65-69 |  |  |  | 1 | (1.6) |  | 5 | (7.9) |  |  |  |  | 3 | (4.7) |  | 1 | (1.6) |
| 70-74 |  |  |  | 2 | (3.2) |  | 5 | (7.9) |  |  |  |  | 2 | (3.1) |  | 1 | (1.6) |
| 75-79 |  |  |  | 2 | (3.2) |  | 1 | (1.6) |  |  |  |  |  |  |  | 4 | (6.5) |
| 80-84 |  |  |  | 1 | (1.6) |  |  |  |  |  |  |  |  |  |  | 1 | (1.6) |
| 85-89 |  |  |  | 2 | (3.2) |  | 2 | (3.2) |  |  |  |  |  |  |  | 2 | (3.2) |
| 90-94 |  |  |  | 2 | (3.2) |  | 3 | (4.8) |  |  |  |  |  |  |  | 2 | (3.2) |
| 95-99 |  |  |  | 2 | (3.2) |  | 2 | (3.2) |  |  |  |  |  |  |  | 1 | (1.6) |

**Table S1. (continued)**

| Surgical volume | Esophagus | |  | Stomach | |  | Colorectum | |  | Pancreas | |  | Lung | |  | Breast | |
| --- | --- | --- | --- | --- | --- | --- | --- | --- | --- | --- | --- | --- | --- | --- | --- | --- | --- |
|  | n | (%) |  | n | (%) |  | n | (%) |  | n | (%) |  | n | (%) |  | n | (%) |
| 100-104 |  |  |  | 2 | (3.2) |  | 2 | (3.2) |  |  |  |  | 1 | (1.6) |  | 2 | (3.2) |
| 105-109 |  |  |  |  |  |  | 1 | (1.6) |  |  |  |  | 2 | (3.1) |  | 1 | (1.6) |
| 110-114 |  |  |  | 2 | (3.2) |  |  |  |  |  |  |  |  |  |  | 1 | (1.6) |
| 115-119 |  |  |  | 1 | (1.6) |  | 3 | (4.8) |  |  |  |  |  |  |  |  |  |
| 120-124 |  |  |  |  |  |  | 2 | (3.2) |  |  |  |  |  |  |  |  |  |
| 125-129 |  |  |  | 1 | (1.6) |  | 2 | (3.2) |  |  |  |  |  |  |  | 1 | (1.6) |
| 130-134 |  |  |  |  |  |  | 3 | (4.8) |  |  |  |  |  |  |  |  |  |
| 135-139 |  |  |  |  |  |  |  |  |  |  |  |  |  |  |  |  |  |
| 140-144 |  |  |  |  |  |  |  |  |  |  |  |  | 1 | (1.6) |  |  |  |
| 145-149 |  |  |  |  |  |  |  |  |  |  |  |  |  |  |  |  |  |
| 150-154 |  |  |  | 1 | (1.6) |  |  |  |  |  |  |  |  |  |  |  |  |
| 155-159 |  |  |  |  |  |  | 1 | (1.6) |  |  |  |  |  |  |  | 1 | (1.6) |
| 160-164 |  |  |  |  |  |  |  |  |  |  |  |  | 1 | (1.6) |  |  |  |
| 165-169 |  |  |  |  |  |  |  |  |  |  |  |  |  |  |  |  |  |
| 170-174 |  |  |  |  |  |  |  |  |  |  |  |  |  |  |  |  |  |
| 175-179 |  |  |  |  |  |  | 1 | (1.6) |  |  |  |  |  |  |  | 1 | (1.6) |
| 180-184 |  |  |  |  |  |  | 1 | (1.6) |  |  |  |  |  |  |  |  |  |
| 185-189 |  |  |  |  |  |  |  |  |  |  |  |  |  |  |  |  |  |
| 190-194 |  |  |  |  |  |  |  |  |  |  |  |  |  |  |  |  |  |
| 195-199 |  |  |  |  |  |  | 1 | (1.6) |  |  |  |  |  |  |  | 1 | (1.6) |

**Table S2. Distribution of patients according to the surgical volume category**

| Surgical volume | Esophagus | |  | Stomach | |  | Colorectum | |  | Pancreas | |  | Lung | |  | Breast | |
| --- | --- | --- | --- | --- | --- | --- | --- | --- | --- | --- | --- | --- | --- | --- | --- | --- | --- |
|  | n | (%) |  | n | (%) |  | n | (%) |  | n | (%) |  | n | (%) |  | n | (%) |
| 0-4 | 372 | (16.8) |  | 22 | (0.1) |  | 13 | (0.1) |  | 396 | (19.5) |  | 128 | (1.4) |  | 46 | (0.3) |
| 5-9 | 299 | (13.5) |  | 118 | (0.8) |  | 103 | (0.5) |  | 542 | (26.6) |  | 99 | (1.1) |  | 292 | (2.0) |
| 10-14 |  |  |  | 109 | (0.7) |  | 121 | (0.6) |  | 402 | (19.8) |  | 243 | (2.7) |  |  |  |
| 15-19 | 166 | (7.5) |  | 511 | (3.4) |  | 165 | (0.8) |  |  |  |  | 387 | (4.4) |  | 153 | (1.0) |
| 20-24 | 215 | (9.7) |  | 416 | (2.8) |  | 115 | (0.5) |  | 242 | (11.9) |  | 842 | (9.5) |  | 319 | (2.2) |
| 25-29 | 144 | (6.5) |  | 628 | (4.2) |  | 140 | (0.6) |  | 127 | (6.2) |  | 379 | (4.3) |  | 373 | (2.5) |
| 30-34 |  |  |  | 451 | (3.0) |  | 596 | (2.8) |  | 326 | (16.0) |  | 623 | (7.0) |  | 1205 | (8.2) |
| 35-39 | 189 | (8.5) |  | 720 | (4.8) |  | 492 | (2.3) |  |  |  |  | 189 | (2.1) |  | 315 | (2.1) |
| 40-44 |  |  |  | 1169 | (7.8) |  | 391 | (1.8) |  |  |  |  | 417 | (4.7) |  | 600 | (4.1) |
| 45-49 | 244 | (11.0) |  | 656 | (4.4) |  | 422 | (2.0) |  |  |  |  |  |  |  | 215 | (1.5) |
| 50-54 |  |  |  | 731 | (4.9) |  | 690 | (3.2) |  |  |  |  | 501 | (5.6) |  |  |  |
| 55-59 | 582 | (26.3) |  | 812 | (5.4) |  | 1035 | (4.8) |  |  |  |  |  |  |  | 554 | (3.8) |
| 60-64 |  |  |  |  |  |  |  |  |  |  |  |  | 306 | (3.4) |  | 613 | (4.2) |
| 65-69 |  |  |  | 325 | (2.2) |  | 1564 | (7.3) |  |  |  |  | 983 | (11.1) |  | 318 | (2.2) |
| 70-74 |  |  |  | 647 | (4.3) |  | 1664 | (7.7) |  |  |  |  | 705 | (7.9) |  | 343 | (2.3) |
| 75-79 |  |  |  | 722 | (4.8) |  | 267 | (1.2) |  |  |  |  |  |  |  | 1471 | (10.0) |
| 80-84 |  |  |  | 373 | (2.5) |  |  |  |  |  |  |  |  |  |  | 387 | (2.6) |
| 85-89 |  |  |  | 847 | (5.6) |  | 819 | (3.8) |  |  |  |  |  |  |  | 853 | (5.8) |
| 90-94 |  |  |  | 883 | (5.9) |  | 1311 | (6.1) |  |  |  |  |  |  |  | 902 | (6.1) |
| 95-99 |  |  |  | 918 | (6.1) |  | 885 | (4.1) |  |  |  |  |  |  |  | 465 | (3.2) |

**Table S2. (continued)**

| Surgical volume | Esophagus | |  | Stomach | |  | Colorectum | |  | Pancreas | |  | Lung | |  | Breast | |
| --- | --- | --- | --- | --- | --- | --- | --- | --- | --- | --- | --- | --- | --- | --- | --- | --- | --- |
|  | n | (%) |  | n | (%) |  | n | (%) |  | n | (%) |  | n | (%) |  | n | (%) |
| 100-104 |  |  |  | 989 | (6.6) |  | 952 | (4.4) |  |  |  |  | 509 | (5.7) |  | 988 | (6.7) |
| 105-109 |  |  |  |  |  |  | 501 | (2.3) |  |  |  |  | 1057 | (11.9) |  | 509 | (3.5) |
| 110-114 |  |  |  | 1093 | (7.3) |  |  |  |  |  |  |  |  |  |  | 548 | (3.7) |
| 115-119 |  |  |  | 564 | (3.8) |  | 1678 | (7.8) |  |  |  |  |  |  |  |  |  |
| 120-124 |  |  |  |  |  |  | 1178 | (5.5) |  |  |  |  |  |  |  |  |  |
| 125-129 |  |  |  | 603 | (4.0) |  | 1204 | (5.6) |  |  |  |  |  |  |  | 622 | (4.2) |
| 130-134 |  |  |  |  |  |  | 1895 | (8.8) |  |  |  |  |  |  |  |  |  |
| 135-139 |  |  |  |  |  |  |  |  |  |  |  |  |  |  |  |  |  |
| 140-144 |  |  |  |  |  |  |  |  |  |  |  |  | 704 | (7.9) |  |  |  |
| 145-149 |  |  |  |  |  |  |  |  |  |  |  |  |  |  |  |  |  |
| 150-154 |  |  |  | 733 | (4.9) |  |  |  |  |  |  |  |  |  |  |  |  |
| 155-159 |  |  |  |  |  |  | 736 | (3.4) |  |  |  |  |  |  |  | 768 | (5.2) |
| 160-164 |  |  |  |  |  |  |  |  |  |  |  |  | 800 | (9.0) |  |  |  |
| 165-169 |  |  |  |  |  |  |  |  |  |  |  |  |  |  |  |  |  |
| 170-174 |  |  |  |  |  |  |  |  |  |  |  |  |  |  |  |  |  |
| 175-179 |  |  |  |  |  |  | 819 | (3.8) |  |  |  |  |  |  |  | 878 | (6.0) |
| 180-184 |  |  |  |  |  |  | 849 | (3.9) |  |  |  |  |  |  |  |  |  |
| 185-189 |  |  |  |  |  |  |  |  |  |  |  |  |  |  |  |  |  |
| 190-194 |  |  |  |  |  |  |  |  |  |  |  |  |  |  |  |  |  |
| 195-199 |  |  |  |  |  |  | 940 | (4.4) |  |  |  |  |  |  |  | 962 | (6.5) |

**Table S3 Mean age of patients according to the surgical volume category**

| Surgical volume | Esophagus | |  | Stomach | |  | Colorectum | |  | Pancreas | |  | Lung | |  | Breast | |
| --- | --- | --- | --- | --- | --- | --- | --- | --- | --- | --- | --- | --- | --- | --- | --- | --- | --- |
|  | Mean | SD |  | Mean | SD |  | Mean | SD |  | Mean | SD |  | Mean | SD |  | Mean | SD |
| 0-4 | 66.2 | (8.6) |  | 70.4 | (7.8) |  | 75.4 | (6.9) |  | 68.6 | (8.7) |  | 69.6 | (8.7) |  | 66.0 | (11.2) |
| 5-9 | 65.1 | (7.9) |  | 69.7 | (9.0) |  | 68.6 | (10.1) |  | 68.1 | (8.7) |  | 69.1 | (9.0) |  | 61.5 | (12.8) |
| 10-14 |  |  |  | 69.0 | (10.3) |  | 69.1 | (10.1) |  | 67.9 | (9.7) |  | 70.1 | (8.4) |  |  |  |
| 15-19 | 66.0 | (8.2) |  | 68.7 | (9.1) |  | 68.3 | (9.9) |  |  |  |  | 69.2 | (8.5) |  | 60.8 | (13.2) |
| 20-24 | 65.6 | (8.3) |  | 67.9 | (9.7) |  | 69.1 | (8.1) |  | 68.3 | (8.2) |  | 68.6 | (8.6) |  | 62.7 | (12.1) |
| 25-29 | 65.9 | (7.3) |  | 68.5 | (9.3) |  | 68.8 | (10.2) |  | 67.8 | (9.1) |  | 68.4 | (9.0) |  | 59.8 | (12.2) |
| 30-34 |  |  |  | 67.5 | (9.8) |  | 68.9 | (9.8) |  | 65.8 | (9.9) |  | 68.3 | (9.1) |  | 59.8 | (12.2) |
| 35-39 | 62.8 | (7.8) |  | 67.7 | (9.7) |  | 69.1 | (9.7) |  |  |  |  | 68.7 | (7.7) |  | 60.2 | (12.5) |
| 40-44 |  |  |  | 67.9 | (10.3) |  | 67.6 | (9.8) |  |  |  |  | 68.5 | (9.0) |  | 61.6 | (12.5) |
| 45-49 | 65.3 | (8.5) |  | 66.9 | (9.5) |  | 67.8 | (10.4) |  |  |  |  |  |  |  | 59.8 | (13.8) |
| 50-54 |  |  |  | 67.5 | (9.9) |  | 67.4 | (9.7) |  |  |  |  | 67.6 | (9.2) |  |  |  |
| 55-59 | 65.2 | (7.9) |  | 66.5 | (10.7) |  | 68.2 | (9.5) |  |  |  |  |  |  |  | 58.7 | (12.8) |
| 60-64 |  |  |  |  |  |  |  |  |  |  |  |  | 67.7 | (8.2) |  | 56.1 | (13.0) |
| 65-69 |  |  |  | 69.0 | (9.5) |  | 68.1 | (9.9) |  |  |  |  | 67.6 | (9.3) |  | 58.6 | (12.4) |
| 70-74 |  |  |  | 68.2 | (10.1) |  | 68.4 | (9.7) |  |  |  |  | 67.3 | (9.8) |  | 59.7 | (13.1) |
| 75-79 |  |  |  | 67.3 | (10.4) |  | 68.9 | (9.5) |  |  |  |  |  |  |  | 60.2 | (12.7) |
| 80-84 |  |  |  | 66.7 | (9.7) |  |  |  |  |  |  |  |  |  |  | 58.2 | (12.9) |
| 85-89 |  |  |  | 65.2 | (10.5) |  | 67.6 | (9.7) |  |  |  |  |  |  |  | 59.1 | (12.3) |
| 90-94 |  |  |  | 66.7 | (9.9) |  | 65.8 | (10.4) |  |  |  |  |  |  |  | 57.7 | (12.7) |
| 95-99 |  |  |  | 66.3 | (10.2) |  | 68.8 | (9.8) |  |  |  |  |  |  |  | 57.5 | (12.2) |

**Table S3 (continued)**

| Surgical volume | Esophagus | |  | Stomach | |  | Colorectum | |  | Pancreas | |  | Lung | |  | Breast | |
| --- | --- | --- | --- | --- | --- | --- | --- | --- | --- | --- | --- | --- | --- | --- | --- | --- | --- |
|  | Mean | SD |  | Mean | SD |  | Mean | SD |  | Mean | SD |  | Mean | SD |  | Mean | SD |
| 100-104 |  |  |  | 67.3 | (9.9) |  | 67.3 | (10.0) |  |  |  |  | 67.7 | (9.0) |  | 58.8 | (12.6) |
| 105-109 |  |  |  |  |  |  | 68.0 | (9.7) |  |  |  |  | 67.6 | (9.5) |  | 60.7 | (12.2) |
| 110-114 |  |  |  | 65.5 | (11.4) |  |  |  |  |  |  |  |  |  |  | 59.8 | (12.3) |
| 115-119 |  |  |  | 67.7 | (10.1) |  | 67.4 | (9.8) |  |  |  |  |  |  |  |  |  |
| 120-124 |  |  |  |  |  |  | 66.5 | (10.4) |  |  |  |  |  |  |  |  |  |
| 125-129 |  |  |  | 66.8 | (10.0) |  | 67.0 | (9.6) |  |  |  |  |  |  |  | 56.1 | (12.6) |
| 130-134 |  |  |  |  |  |  | 67.3 | (9.8) |  |  |  |  |  |  |  |  |  |
| 135-139 |  |  |  |  |  |  |  |  |  |  |  |  |  |  |  |  |  |
| 140-144 |  |  |  |  |  |  |  |  |  |  |  |  | 65.8 | (9.4) |  |  |  |
| 145-149 |  |  |  |  |  |  |  |  |  |  |  |  |  |  |  |  |  |
| 150-154 |  |  |  | 67.2 | (9.7) |  |  |  |  |  |  |  |  |  |  |  |  |
| 155-159 |  |  |  |  |  |  | 68.5 | (9.8) |  |  |  |  |  |  |  | 55.5 | (12.3) |
| 160-164 |  |  |  |  |  |  |  |  |  |  |  |  | 67.0 | (8.6) |  |  |  |
| 165-169 |  |  |  |  |  |  |  |  |  |  |  |  |  |  |  |  |  |
| 170-174 |  |  |  |  |  |  |  |  |  |  |  |  |  |  |  |  |  |
| 175-179 |  |  |  |  |  |  | 68.4 | (9.5) |  |  |  |  |  |  |  | 55.7 | (11.6) |
| 180-184 |  |  |  |  |  |  | 67.2 | (10.8) |  |  |  |  |  |  |  |  |  |
| 185-189 |  |  |  |  |  |  |  |  |  |  |  |  |  |  |  |  |  |
| 190-194 |  |  |  |  |  |  |  |  |  |  |  |  |  |  |  |  |  |
| 195-199 |  |  |  |  |  |  | 65.0 | (10.7) |  |  |  |  |  |  |  | 56.9 | (12.5) |

**Table S4 Proportion of patients with localized stage according to the surgical volume category**

| Surgical volume | Esophagus | |  | Stomach | |  | Colorectum | |  | Pancreas | |  | Lung | |  | Breast | |
| --- | --- | --- | --- | --- | --- | --- | --- | --- | --- | --- | --- | --- | --- | --- | --- | --- | --- |
|  | n | (%) |  | n | (%) |  | n | (%) |  | n | (%) |  | n | (%) |  | n | (%) |
| 0-4 | 122 | (32.8) |  | 1 | (4.6) |  | 2 | (15.4) |  | 52 | (13.1) |  | 54 | (42.2) |  | 23 | (50.0) |
| 5-9 | 84 | (28.1) |  | 26 | (22.0) |  | 14 | (13.6) |  | 85 | (15.7) |  | 74 | (74.8) |  | 182 | (62.3) |
| 10-14 |  |  |  | 28 | (25.7) |  | 38 | (31.4) |  | 49 | (12.2) |  | 136 | (56.0) |  |  |  |
| 15-19 | 48 | (28.9) |  | 213 | (41.7) |  | 56 | (33.9) |  |  |  |  | 216 | (55.8) |  | 103 | (67.3) |
| 20-24 | 69 | (32.1) |  | 182 | (43.8) |  | 33 | (28.7) |  | 30 | (12.4) |  | 504 | (59.9) |  | 203 | (63.6) |
| 25-29 | 35 | (24.3) |  | 250 | (39.8) |  | 69 | (49.3) |  | 25 | (19.7) |  | 213 | (56.2) |  | 253 | (67.8) |
| 30-34 |  |  |  | 231 | (51.2) |  | 252 | (42.3) |  | 41 | (12.6) |  | 398 | (63.9) |  | 831 | (69.0) |
| 35-39 | 37 | (19.6) |  | 331 | (46.0) |  | 233 | (47.4) |  |  |  |  | 141 | (74.6) |  | 202 | (64.1) |
| 40-44 |  |  |  | 600 | (51.3) |  | 158 | (40.4) |  |  |  |  | 317 | (76.0) |  | 394 | (65.7) |
| 45-49 | 37 | (15.2) |  | 321 | (48.9) |  | 191 | (45.3) |  |  |  |  |  |  |  | 153 | (71.2) |
| 50-54 |  |  |  | 311 | (42.5) |  | 354 | (51.3) |  |  |  |  | 301 | (60.1) |  |  |  |
| 55-59 | 155 | (26.6) |  | 371 | (45.7) |  | 461 | (44.5) |  |  |  |  |  |  |  | 393 | (70.9) |
| 60-64 |  |  |  |  |  |  |  |  |  |  |  |  | 189 | (61.8) |  | 425 | (69.3) |
| 65-69 |  |  |  | 155 | (47.7) |  | 683 | (43.7) |  |  |  |  | 642 | (65.3) |  | 181 | (56.9) |
| 70-74 |  |  |  | 303 | (46.8) |  | 657 | (39.5) |  |  |  |  | 465 | (66.0) |  | 246 | (71.7) |
| 75-79 |  |  |  | 340 | (47.1) |  | 89 | (33.3) |  |  |  |  |  |  |  | 976 | (66.4) |
| 80-84 |  |  |  | 213 | (57.1) |  |  |  |  |  |  |  |  |  |  | 240 | (62.0) |
| 85-89 |  |  |  | 554 | (65.4) |  | 375 | (45.8) |  |  |  |  |  |  |  | 637 | (74.7) |
| 90-94 |  |  |  | 439 | (49.7) |  | 691 | (52.7) |  |  |  |  |  |  |  | 580 | (64.3) |
| 95-99 |  |  |  | 462 | (50.3) |  | 356 | (40.2) |  |  |  |  |  |  |  | 286 | (61.5) |

**Table S4 (continued)**

| Surgical volume | Esophagus | |  | Stomach | |  | Colorectum | |  | Pancreas | |  | Lung | |  | Breast | |
| --- | --- | --- | --- | --- | --- | --- | --- | --- | --- | --- | --- | --- | --- | --- | --- | --- | --- |
|  | n | (%) |  | n | (%) |  | n | (%) |  | n | (%) |  | n | (%) |  | n | (%) |
| 100-104 |  |  |  | 503 | (50.9) |  | 460 | (48.3) |  |  |  |  | 293 | (57.6) |  | 624 | (63.2) |
| 105-109 |  |  |  |  |  |  | 198 | (39.5) |  |  |  |  | 657 | (62.2) |  | 323 | (63.5) |
| 110-114 |  |  |  | 549 | (50.2) |  |  |  |  |  |  |  |  |  |  | 368 | (67.2) |
| 115-119 |  |  |  | 295 | (52.3) |  | 735 | (43.8) |  |  |  |  |  |  |  |  |  |
| 120-124 |  |  |  |  |  |  | 492 | (41.8) |  |  |  |  |  |  |  |  |  |
| 125-129 |  |  |  | 351 | (58.2) |  | 565 | (46.9) |  |  |  |  |  |  |  | 478 | (76.9) |
| 130-134 |  |  |  |  |  |  | 819 | (43.2) |  |  |  |  |  |  |  |  |  |
| 135-139 |  |  |  |  |  |  |  |  |  |  |  |  |  |  |  |  |  |
| 140-144 |  |  |  |  |  |  |  |  |  |  |  |  | 493 | (70.0) |  |  |  |
| 145-149 |  |  |  |  |  |  |  |  |  |  |  |  |  |  |  |  |  |
| 150-154 |  |  |  | 394 | (53.8) |  |  |  |  |  |  |  |  |  |  |  |  |
| 155-159 |  |  |  |  |  |  | 376 | (51.1) |  |  |  |  |  |  |  | 498 | (64.8) |
| 160-164 |  |  |  |  |  |  |  |  |  |  |  |  | 540 | (67.5) |  |  |  |
| 165-169 |  |  |  |  |  |  |  |  |  |  |  |  |  |  |  |  |  |
| 170-174 |  |  |  |  |  |  |  |  |  |  |  |  |  |  |  |  |  |
| 175-179 |  |  |  |  |  |  | 359 | (43.8) |  |  |  |  |  |  |  | 572 | (65.2) |
| 180-184 |  |  |  |  |  |  | 356 | (41.9) |  |  |  |  |  |  |  |  |  |
| 185-189 |  |  |  |  |  |  |  |  |  |  |  |  |  |  |  |  |  |
| 190-194 |  |  |  |  |  |  |  |  |  |  |  |  |  |  |  |  |  |
| 195-199 |  |  |  |  |  |  | 410 | (43.6) |  |  |  |  |  |  |  | 522 | (54.3) |

**Table S5. Adjusted hazard ratios and adjusted 5-year survival probability by surgical volume category**

| Surgical volume | Esophagus | | Stomach | | Colorectum | | Pancreas | | Lung | | Breast | |
| --- | --- | --- | --- | --- | --- | --- | --- | --- | --- | --- | --- | --- |
|  | aHR (95%CI) | Survival^†^ | aHR (95%CI) | Survival | aHR (95%CI) | Survival | aHR (95%CI) | Survival | aHR (95%CI) | Survival | aHR (95%CI) | Survival |
| 0-4 | 1.79(1.35-2.37) | 34.0 | 5.49(4.52-6.67) | 18.6 | 4.40(3.69-5.24) | 22.7 | 1.73(1.15-2.58) | 8.3 | 2.64(1.97-3.53) | 43.0 | 1.81(1.00-3.26) | 86.2 |
| 5-9 | 1.45(1.16-1.80) | 41.8 | 3.09(2.57-3.70) | 38.8 | 2.26(2.00-2.55) | 46.8 | 1.28(0.86-1.92) | 15.7 | 1.19(0.75-1.89) | 68.2 | 1.51(1.08-2.10) | 88.3 |
| 10-14 |  |  | 2.25(1.83-2.75) | 50.2 | 1.88(1.54-2.30) | 53.0 | 1.27(0.86-1.88) | 16.0 | 1.45(1.22-1.73) | 62.8 |  |  |
| 15-19 | 1.46(1.01-2.11) | 41.4 | 2.29(2.00-2.61) | 49.6 | 1.90(0.91-3.98) | 52.6 |  |  | 1.56(1.36-1.79) | 60.7 | 1.72(0.70-4.23) | 86.8 |
| 20-24 | 1.61(1.27-2.04) | 37.9 | 1.52(1.21-1.91) | 62.8 | 1.72(1.53-1.92) | 56.1 | 1.05(0.70-1.58) | 21.9 | 1.39(1.18-1.63) | 64.1 | 0.80(0.61-1.05) | 93.6 |
| 25-29 | 1.11(0.80-1.53) | 51.4 | 1.62(1.37-1.92) | 60.8 | 0.72(0.62-0.85) | 78.4 | 1.38(0.96-1.98) | 13.7 | 1.27(1.05-1.54) | 66.6 | 1.04(0.78-1.40) | 91.8 |
| 30-34 |  |  | 1.27(1.02-1.58) | 67.7 | 1.25(1.10-1.43) | 65.6 | 1.00 | 23.7 | 1.07(0.94-1.21) | 71.0 | 0.88(0.58-1.34) | 93.0 |
| 35-39 | 1.04(0.85-1.27) | 53.4 | 1.39(1.14-1.69) | 65.3 | 1.19(0.95-1.48) | 67.1 |  |  | 0.92(0.78-1.08) | 74.4 | 1.05(0.81-1.35) | 91.8 |
| 40-44 |  |  | 1.31(1.16-1.47) | 67.0 | 0.83(0.72-0.96) | 75.7 |  |  | 1.12(0.93-1.36) | 69.8 | 1.04(0.82-1.31) | 91.8 |
| 45-49 | 1.22(0.89-1.68) | 47.9 | 1.26(0.92-1.73) | 67.9 | 0.86(0.64-1.15) | 74.9 |  |  |  |  | 0.70(0.53-0.92) | 94.4 |
| 50-54 |  |  | 1.73(1.51-1.99) | 58.8 | 0.96(0.88-1.04) | 72.5 |  |  | 1.07(0.92-1.26) | 70.9 |  |  |
| 55-59 | 1.00 | 54.7 | 1.31(1.08-1.59) | 66.9 | 1.07(0.93-1.23) | 69.7 |  |  |  |  | 0.99(0.72-1.36) | 92.2 |
| 60-64 |  |  |  |  |  |  |  |  | 0.76(0.63-0.91) | 78.5 | 0.88(0.52-1.49) | 93.0 |
| 65-69 |  |  | 1.34(1.13-1.59) | 66.3 | 1.07(0.92-1.25) | 69.7 |  |  | 1.23(1.00-1.53) | 67.4 | 1.05(0.82-1.34) | 91.8 |
| 70-74 |  |  | 1.68(1.34-2.12) | 59.7 | 1.02(0.84-1.25) | 70.9 |  |  | 1.18(0.88-1.58) | 68.5 | 0.79(0.48-1.30) | 93.7 |
| 75-79 |  |  | 1.32(1.14-1.53) | 66.7 | 1.07(0.98-1.16) | 69.8 |  |  |  |  | 0.90(0.67-1.20) | 92.9 |
| 80-84 |  |  | 1.85(1.62-2.11) | 56.8 |  |  |  |  |  |  | 0.77(0.60-0.99) | 93.9 |
| 85-89 |  |  | 1.11(0.80-1.55) | 71.1 | 0.93(0.73-1.19) | 73.1 |  |  |  |  | 1.11(0.74-1.66) | 91.3 |
| 90-94 |  |  | 1.18(1.04-1.34) | 69.6 | 0.79(0.68-0.91) | 76.8 |  |  |  |  | 0.84(0.69-1.01) | 93.4 |
| 95-99 |  |  | 1.08(0.77-1.51) | 71.9 | 1.13(1.01-1.26) | 68.3 |  |  |  |  | 1.09(0.81-1.46) | 91.4 |

**Table S5. (continued)**

| Surgical volume | Esophagus | | Stomach | | Colorectum | | Pancreas | | Lung | | Breast | |
| --- | --- | --- | --- | --- | --- | --- | --- | --- | --- | --- | --- | --- |
|  | aHR (95%CI) | Survival^†^ | aHR (95%CI) | Survival | aHR (95%CI) | Survival | aHR (95%CI) | Survival | aHR (95%CI) | Survival | aHR (95%CI) | Survival |
| 100-104 |  |  | 0.94(0.82-1.09) | 74.9 | 1.12(0.86-1.45) | 68.6 |  |  | 1.23(1.09-1.39) | 67.3 | 0.77(0.58-1.02) | 93.9 |
| 105-109 |  |  |  |  | 0.86(0.68-1.09) | 74.8 |  |  | 1.49(1.26-1.77) | 62.0 | 0.77(0.58-1.02) | 93.9 |
| 110-114 |  |  | 1.50(1.22-1.85) | 63.1 |  |  |  |  |  |  | 1.07(0.93-1.23) | 91.6 |
| 115-119 |  |  | 0.72(0.59-0.88) | 80.2 | 0.89(0.75-1.07) | 74.0 |  |  |  |  |  |  |
| 120-124 |  |  |  |  | 1.03(0.93-1.15) | 70.6 |  |  |  |  |  |  |
| 125-129 |  |  | 1.53(1.37-1.71) | 62.6 | 0.94(0.71-1.25) | 72.8 |  |  |  |  | 0.71(0.55-0.92) | 94.3 |
| 130-134 |  |  |  |  | 0.97(0.75-1.24) | 72.2 |  |  |  |  |  |  |
| 135-139 |  |  |  |  |  |  |  |  |  |  |  |  |
| 140-144 |  |  |  |  |  |  |  |  | 0.68(0.58-0.81) | 80.4 |  |  |
| 145-149 |  |  |  |  |  |  |  |  |  |  |  |  |
| 150-154 |  |  | 1.00 | 73.6 |  |  |  |  |  |  |  |  |
| 155-159 |  |  |  |  | 1.25(1.12-1.39) | 65.6 |  |  |  |  | 0.84(0.64-1.10) | 93.3 |
| 160-164 |  |  |  |  |  |  |  |  | 1.00 | 72.6 |  |  |
| 165-169 |  |  |  |  |  |  |  |  |  |  |  |  |
| 170-174 |  |  |  |  |  |  |  |  |  |  |  |  |
| 175-179 |  |  |  |  | 0.77(0.67-0.90) | 77.0 |  |  |  |  | 0.65(0.53-0.79) | 94.8 |
| 180-184 |  |  |  |  | 0.87(0.73-1.03) | 74.6 |  |  |  |  |  |  |
| 185-189 |  |  |  |  |  |  |  |  |  |  |  |  |
| 190-194 |  |  |  |  |  |  |  |  |  |  |  |  |
| 195-199 |  |  |  |  | 1.00 | 71.4 |  |  |  |  | 1.00 | 92.1 |

Note. aHR: adjusted hazard ratios. CI: confidence interval. † Survival indicates the adjusted 5-year survival probability estimated using a multivariable Cox proportional hazard model. The hazard ratios and 5-year survival probability were adjusted for sex, age, stage, surgical procedure type, existence of residual tumor, reception of chemo/hormone therapy, reception of radiation therapy, residential area, and years of diagnosis.

**Figure S1 Trends of adjusted 5-year survival probability per surgical volume category and the suggested minimum surgical volume among participants with localized or regional stage of cancers.**


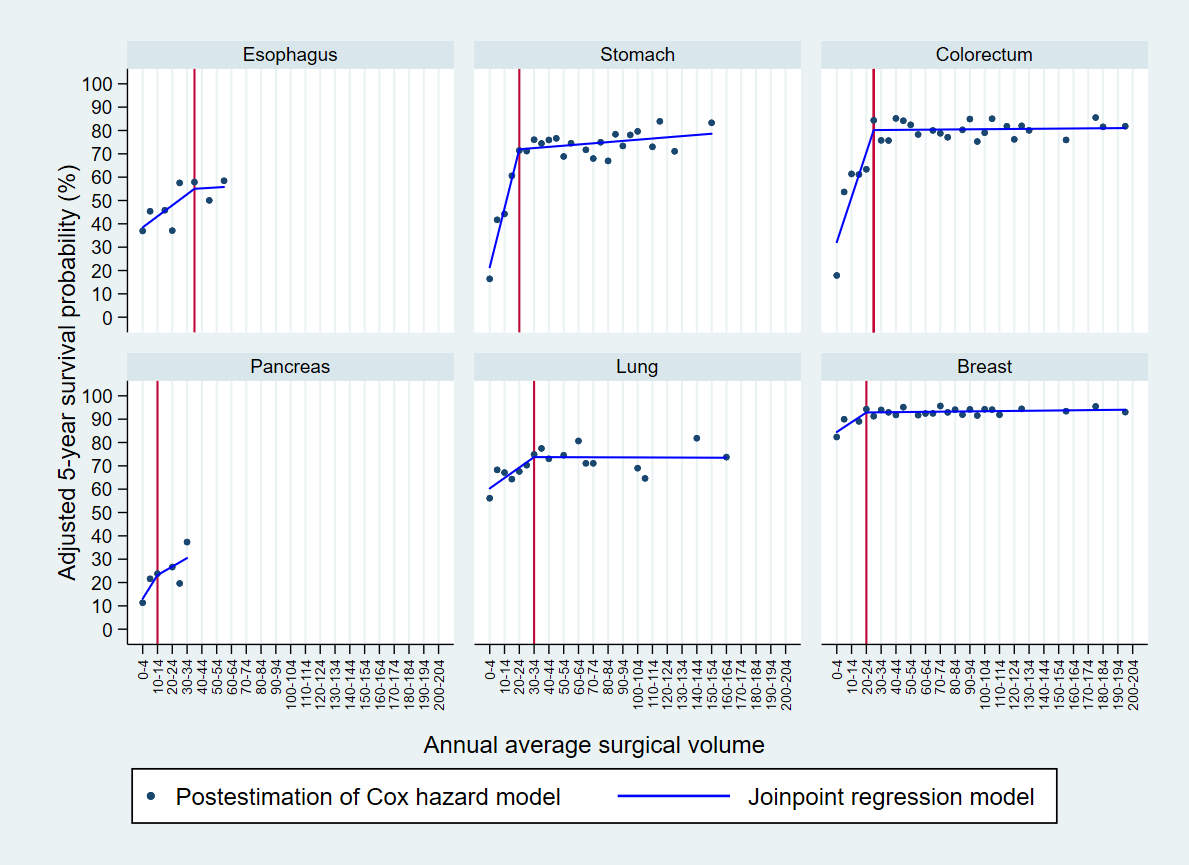


Note. We described the adjusted 5-year survival probability per annual surgical volume category estimated by a multivariable Cox proportional hazard model. Based on the association between surgical volume category and adjusted 5-year survival probability, we described the linear relationship and identified the minimum surgical volume using the joinpoint regression model. Red vertical lines indicate the suggested minimum surgical volume.
